# Treatment of Fabella syndrome with arthroscopic fabellectomy: a case series and literature review

**DOI:** 10.1186/s12891-021-04630-w

**Published:** 2021-08-30

**Authors:** Shuo-Po Weng, Tsung-Mu Wu, Chi-Sheng Chien, Sheng-Hui Lin

**Affiliations:** 1 Orthopedic Department, Kaohsiung Veterans General Hospital, No.386, Dazhong 1st Rd., Zuoying Dist., Kaohsiung City, Taiwan (Republic of China); 2 Orthopedic Department, Chi-Mei Medical Center, No.901, Zhonghua Rd., Yongkang Dist., Tainan City, Taiwan (Republic of China)

**Keywords:** Fabella syndrome, Fabella impingement, All-arthroscopic fabellectomy, Posterolateral knee pain, Fabella chondromalacia, Fabella osteoarthritis

## Abstract

**Background:**

The fabella is a sesamoid bone in the posterolateral capsule of the human knee joint. In quadrupedal mammals, the fabella is believed to have a role similar to the patella in redirecting extension forces of the knee joint from one point to another. In bipeds, the fabella is not touching the back of the bent knee, and therefore the role in redirecting forces declines. Posterolateral knee pain can be associated with the irritation between the fabella and lateral femoral condyle, a phenomenon also known as fabella syndrome. In cases that are unresponsive to conservative management, surgical fabellectomy can be a successful treatment option. Among the surgical approaches, open resection is most commonly seen. There are also literature reporting arthroscopic-assisted open resection, but seldom mentioned the all-arthroscopic fabellectomy.

**Case presentation:**

We present 3 patients with a long history (> 12 month) of posterolateral knee pain under suspicion of different pain origins. The diagnosis of fabella impingement was eventually made by ruling out of other causes. All the patients underwent all-arthroscopic fabellectomy for diagnosis and treatment. Investigations of the resected fabella suggested chronic impingement with apparent osteophyte formation and cartilage wearing of the articular side. All patients have been continually followed up at our outpatient department and reported to be pain free after the procedure.

**Conclusions:**

In the patients presenting posterolateral pain, fabella syndrome cannot be ignored due to its relative higher presence in Asian population. In our experience, the all-arthroscopic fabellectomy offers a smaller wound size, less post-operative pain, fewer days of hospitalization and quicker time to rehabilitation for the patients with chronic posterolateral knee pain caused by fabella syndrome.

## Background

The fabella is a sesamoid bone, which is located at the posterolateral capsule and embedded in lateral head of gastrocnemius. The fabella can function similarly to the patella, redirecting the tensile forces generated by the quadriceps, increasing its mechanical advantage. This effect can be larger in quadrupedal mammals, which generally have a more laterally located fabella, compared to bipedal mammals [1]. The presence of fabella varies widely in individuals, ranging from 3 to 87% per knee [2], and it was more commonly observed in Asian, Oceania, and South American populations than European, North American, and African populations [3].

However, presence of the fabella can cause irritation at the posterior side of lateral femoral condyle, producing intermittent posterolateral knee pain due to the compression and shearing force between the fabella and lateral femoral condyle [4]. This phenomenon can be referred to as fabella syndrome. Therefore, when it comes to posterolateral knee pain, fabella syndrome should be considered one of the diagnosis after ruling out other common knee derangement [5–7].

The non-operative treatment of fabella syndrome nowadays includes oral medication, physiotherapy, and local anesthetic agent injection. Local anesthetic agent injection in outpatient department can serve both as diagnostic and therapeutic treatment [1]. However, if all the conservative treatment fails to relieve the symptoms, surgical treatment can be considered. Present surgical treatment of fabella syndrome is the fabellectomy, which can be done by open, arthroscopy-assisted [8], or all-arthroscopic excision [9]. The purpose of our article was to share our experience of the all-arthroscopic fabellectomy.

## Case presentation

### Case 1

**Fig. 1:**
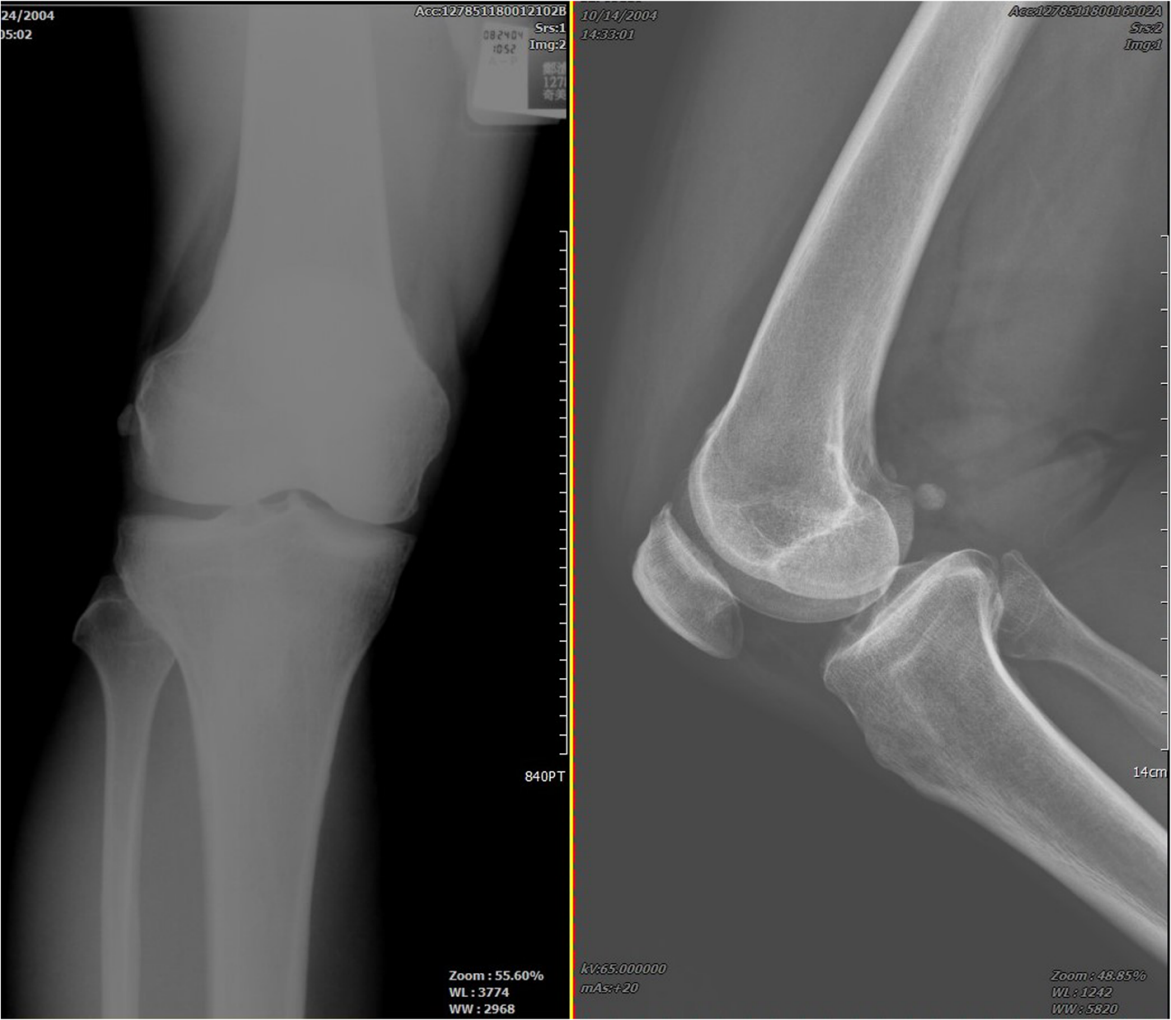
The right knee X-ray showed mild osteoarthritis with a significant fabella bone at the posterolateral side of knee

**Fig. 2:**
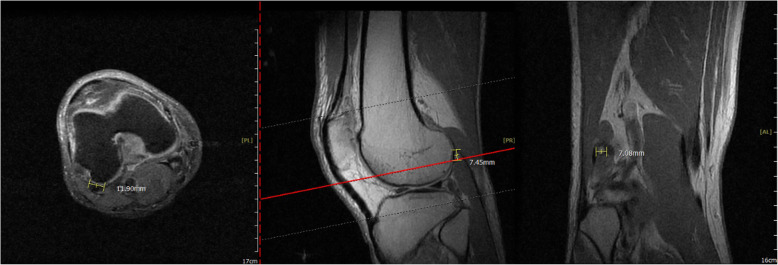
The right knee MRI showed a fabella with subchondral cyst formation in Coronary view

This 58-year-old male patient had suffered from persistent right posterolateral knee pain since a falling down episode with knee hitting the ground about 4 months ago. In our outpatient department, general discomfort among popliteal fossa was mentioned. There was a limitation of active knee extension at the last 10° due to posterolateral knee pain. The plain radiograph of right knee revealed no significant fracture pattern that may cause the discomfort. [Fig. 1] MRI of right knee was then arranged and showed suspicion of lateral meniscus central longitudinal tear, also, a fabella with significant arthritic change posterior to the lateral femoral condyle was noted. [Fig. 2] Under the impression of the right knee lateral meniscus tear and fabella syndrome, the patient received all-arthroscopic fabellectomy and the meniscus debridement.

### Case 2

**Fig. 3:**
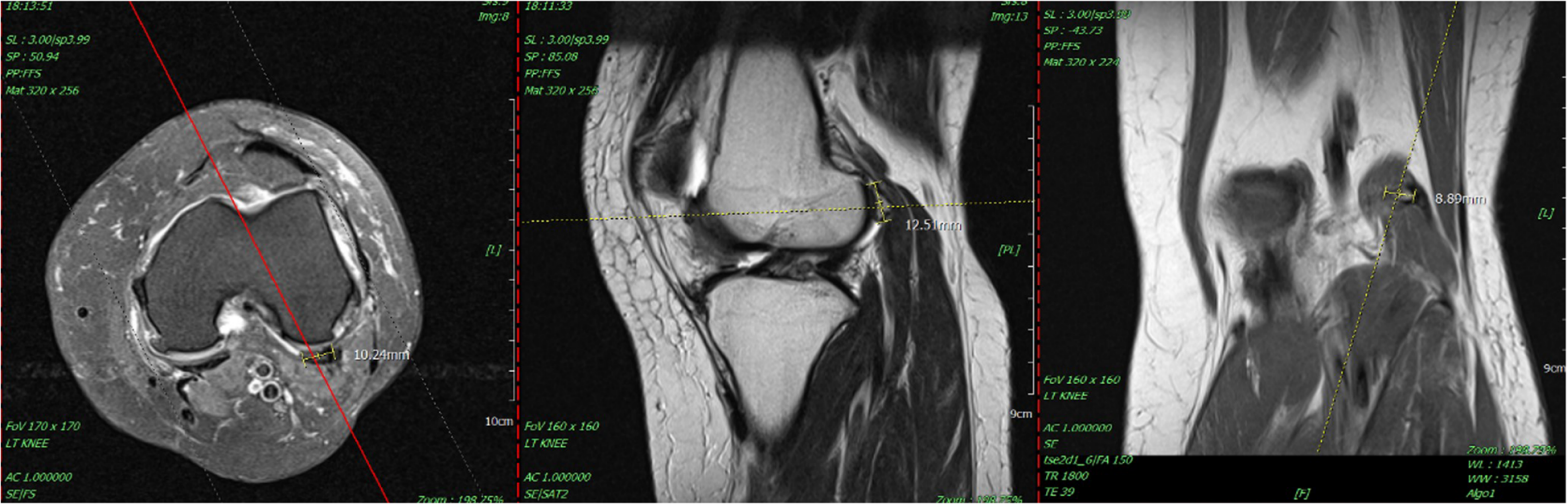
The left knee MRI showed a joint intruded fabella with tear of lateral meniscus in Sagittal view

The 57-year-old woman had been complaining of persistent left knee pain with clicking sensation especially while walking down stairs. The condition had persisted for 3 months, so she came to our outpatient department for evaluation. The physical examination showed pain and tenderness at posterolateral side of knee with exacerbation while performing extension of knee joint. No apparent limitation of range of motion was observed. The McMurray test was negative. The plain radiograph revealed only mild knee osteoarthritis without significant pain source. The MRI was arranged and showed mild degenerative tear of both lateral and medial meniscus. A large fabella up to 12 mm with significant osteophyte formation that articulated with the posterior side of lateral femoral condyle which correlated with the physical examination. [Fig. 3] The patient then underwent all-arthroscopic fabellectomy and meniscus debridement.

### Case 3

**Fig. 4:**
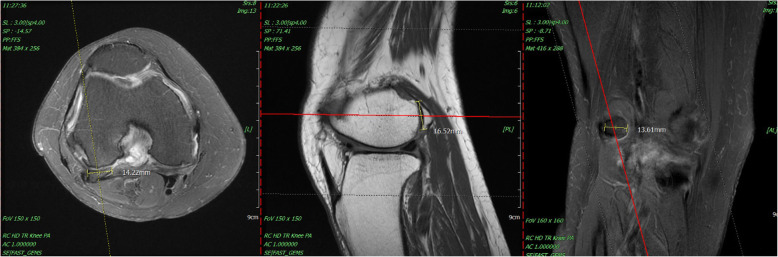
The right knee MRI showed a large fabella bone with subchondral bone edema and peripheral soft tissue effusion

**Fig. 5:**
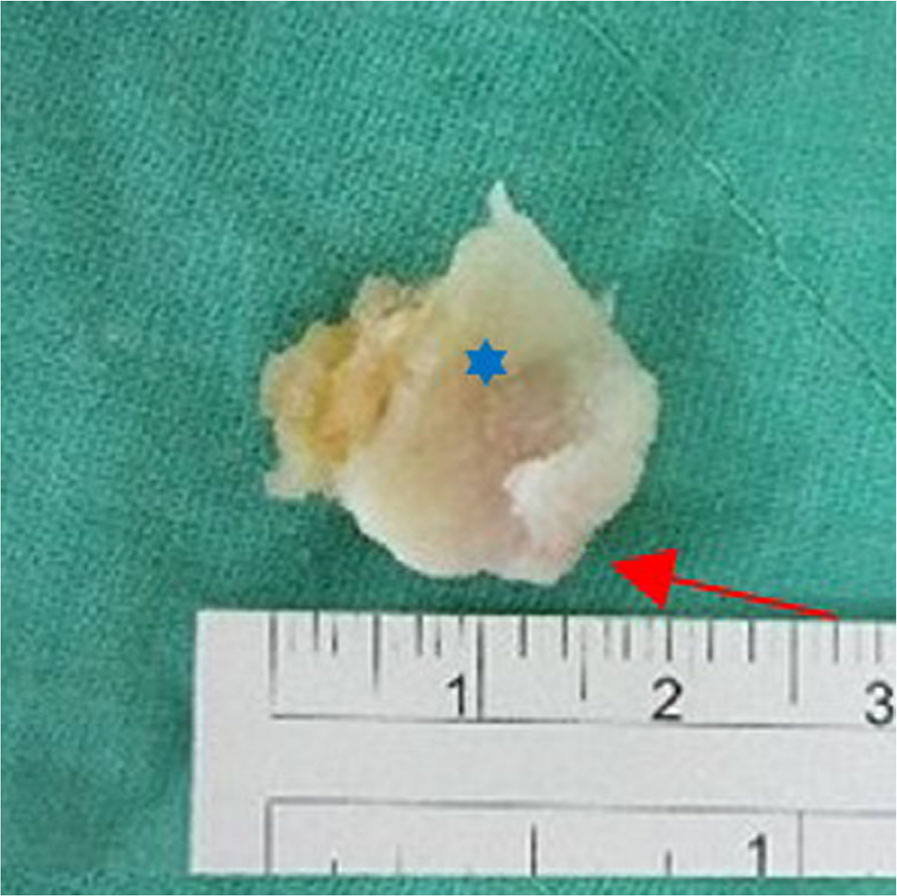
The resected fabella with marginal osteophyte formation(Red arrow) and significant cartilage wear(Blue asterisk) in the middle of bone

This 68-year-old female patient had suffered from chronic right knee pain for more than a year. The physical examination revealed tenderness point at medial joint line and posterolateral fossa. Pain and limited knee range of motion at the last 20° extension was noted. She had to walk with a crutch to lessen the pain of right knee. The plain radiograph and MRI of knee showed moderate osteoarthritis with a large size arthritic change fabella up to 16 mm located at the posterior side of lateral femoral condyle. [Fig. 4] She tried oral medication for pain control and rehabilitation treatment at local clinic but no significant improvement was noted. Thus, we performed the all-arthroscopic fabellectomy. The specimen was an osteochondral fragment as large as 19*17*8 mm with significant osteophyte formation and cartilage wear. [Fig. 5].

### Surgical methods

**Fig. 6:**
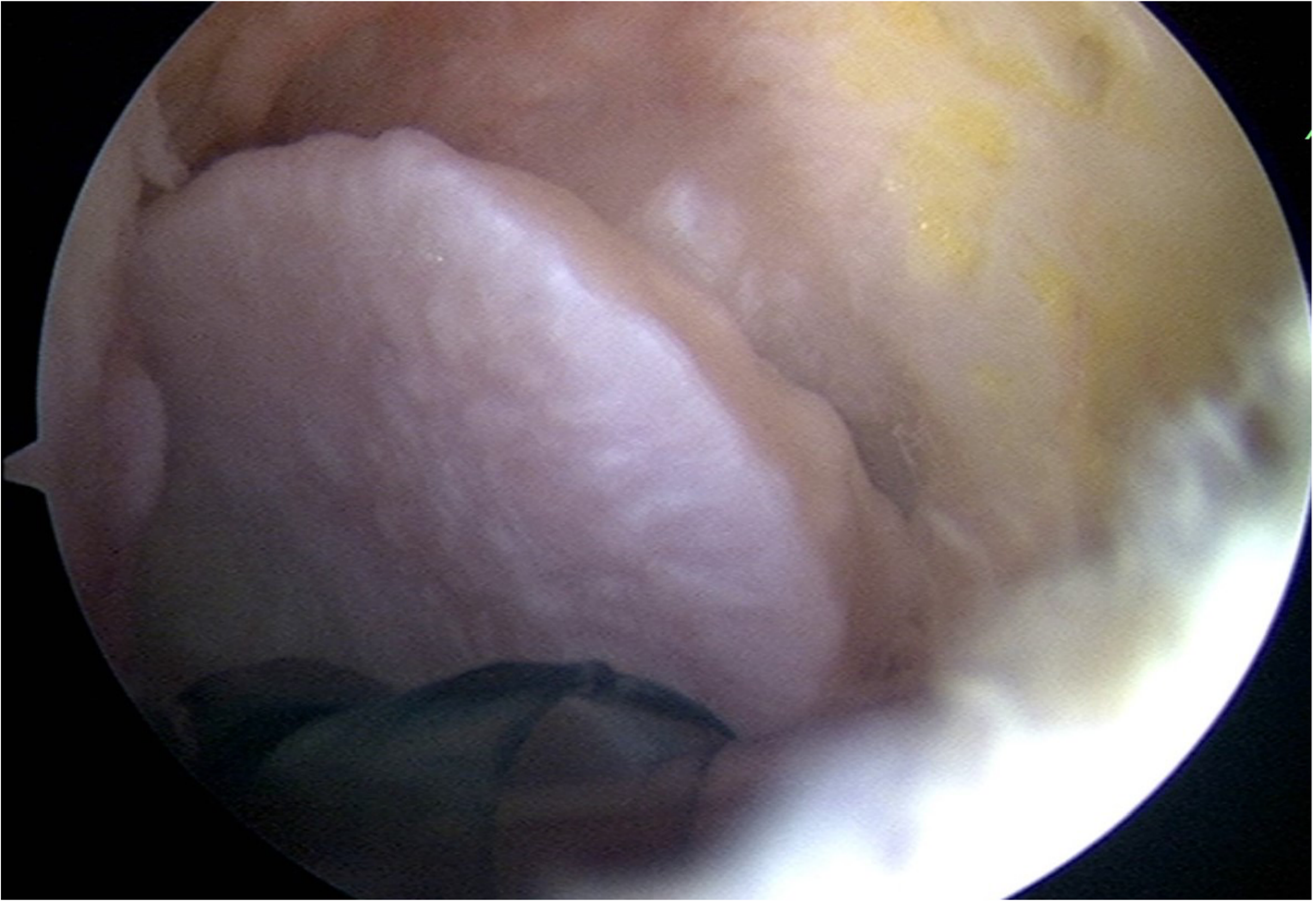
Arthroscopic intraarticular release of the fabella

For every case, the knee was inspected under general anesthesia by Propofol, dosage around 2 mg/kg, with the patient in a supine position. A standard anteromedial, anterolateral are used for the routine arthroscopic examination of the knee joint. The anteromedial and anterolateral portals are placed immediately adjacent to the patellar tendon to allow easy access to the posterior compartments. After flexing the knee to 80°, we inserted the arthroscope via the anterolateral portal, bypassing the intercondylar notch to the posteromedial compartment. Posteromedial portal was made by palpation of the posteromedial region with our digit to locate the arthroscope and double check by the transillumination test. Next, we drew a line along the posterior cortex of fibula and located the point that intersect with the joint line of knee. A spinal needle was inserted from this point and the trajectory was slightly adjusted according to the view from the posteromedial portal, allowing easy access to the posterolateral compartment. The arthroscope was then switched to the posterolateral portal and probe was introduced through the posteromedial portal to locate the fabella. For case of fabella syndrome, the fabella can be identified arthroscopically by simply probing the inner wall of joint. The capsule between the fabella and lateral femoral condyle is usually attenuated due to the prolong shearing force between them that made it softer than the other part of capsule. In the last case, the fabella can even be seen directly from the disrupted capsule. [Fig. 6] After the fabella was located, thermal cautery instrument was used to release the fabella from the lateral head of the gastrocnemius and the surrounding synovium. The posterolateral portal incision is extended if required for retrieval of the fabella.

## Results

After the all-arthroscopic fabellectomy, all patients have been continually followed up at our outpatient department, with the longest case of nearly 11 years.

The patient in case 1 restored his knee range of motion from 0 to 130° without pain during movement. Mild soreness at posterolateral knee was mentioned initially after the surgery, but the discomfort diminished gradually and reported to be VAS 1 compared to VAS 6 pre-operation. The patient in case 2 has sustained mild knee flexion weakness after the surgery, but pain management has significantly improved from VAS 5 to 2. After 2 months of rehabilitation, the patient regained full muscle power with 0–135° knee flexion and pain improvement to VAS 1. Finally, the patient in the last case with the most limited knee range of motion can reach 10–110° knee flexion immediately after the surgery. This can be attributed to the dramatic pain reduction from VAS 6 to 2. At the latest follow up, the patient can walk nearly pain-free without crutch assistant, with the knee range of motion of 0–125°.

## Discussion and conclusions

In Japan, according to Kawashima et al., they observed 66% of 39 Japanese cadavers had presence of fabella [10]. In another study of Chinese population, fabella was noted in 31.25% of people [11]. However, O.F. Egerci et al. described the prevalence of fabella in Turkish was about 22.8% in 500 patients examined by bilateral knee radiographs. The result is quite similar with other Caucasian population [12]. Tudor Sorin et al. reported an even lower incidence of 16.93% in the Romanian [13]. In the studies of Berthaume and Bull, the presence of fabella was most common in Asian populations, followed by those of Oceania, South America, Europe, North America, Africa. Due to the variation of prevalence rate among different races, they assumed that the fabella formation was largely affected by genetics. They also hypothesized that fabella ossification may be controlled by environmental and functional factors, such as mechanical stimuli. This was supported by higher prevalence in males, and an increasing prevalence with age [3].

The fabella syndrome typically presents with posterolateral knee pain, especially during full extension of knee [4]. Applying force on the fabella can exaggerate the pain. Because of the adjacent anatomical relation of fabella and common fibular nerve, the fabella syndrome can present with common fibular nerve palsy, causing foot drop and sensory neuropathy. Tabira et al. observed 102 knees in 51 Japanese cadavers (68.6% fabella presence) and described a significant difference in the diameter of the common fibular nerve between the presence of fabella or not [14].

In individuals who had received total knee replacement, the late onset of fabella syndrome may cause mechanical irritation or compression of posterolateral tissue of the knee. In the case report of Takeshi Kimura et al., a 64-year-old female, who had received total knee arthroplasty (TKA) surgery 8 years ago, had suffered from severe posterolateral pain that prohibited her from the last 30 degree of extension [15]. A large 2 cm fabella was noted in the radiograph. After performing the excision of fabella, the pain relieved and the full range of motion was gradually reached. In order to prevent possible impingement of fabella after TKA, Larson et al. recommended provident excision of the fabella during the TKA procedure [16].

The conservative treatment of fabella syndrome included physiotherapy, activity restriction, medication, and local steroid or analgesic agent injections. J. Tim Zipple reported a case of 44-year male with fabella syndrome had an immediate relief of posterolateral knee pain and 30 degrees increase in knee active flexion via manual therapy. The duration of relief can be more than 16 months [17].

Pyong-Hwa Seol et al. applied radial extracorporeal shock wave therapy(rESWT) as a new treatment for fabella syndrome [18]. Four patients of the report received rESWT(3000 shock waves with 12 Hz delivered at the intensity of 3–5 bar) at the fabella under ultrasoundgraphic guidance. The pain score of the patients decreased and one patient even achieved full knee extension. These effects can last up to 2 months.

Conventional surgical intervention of fabella syndrome was performed by direct approach to the posterolateral aspect in most cases. Matthew T. Provencher et al. presented a surgical technique of fabella excision under the assistance of arthroscopy [8]. It allows surgeons to evaluate the fabella through the arthroscopy, minimize the damage and prevent over-resection of surrounding structures. The long-term studies with large sample sizes are also necessary for evaluation and compares to the non-operative treatment.

Travis J. Dekker et al. reported 11 cases who received arthroscopy-assisted fabllectomy with an average follow up of 2.4 years. The WOMAC total score of the patients significantly improved after receiving the surgery. However, one patient was diagnosed with postoperative arthrofibrosis and required additional adhesiolysis. Therefore, the risks need to be considered and informed before the surgical intervention [4].

However, there are only few studies mentioned all-arthroscopic fabellectomy. Z Dannawi et al. [9] was the first to publish the excision of the fabella using an all-arthroscopic technique. After standard knee examination via anteromedial and anterolateral portal, a long spinal needle was inserted is inserted into the posterolateral compartment using the arthroscopic transillumination from anteromedial portal. A posterolateral portal is created over the spinal needle, allowing easy access to the compartment. The most important thing while locating the proper position of this portal is to avoid common peroneal nerve injury. Keeping the knee flexion and performing soft tissue dissection to the joint as gentle as possible can avoid blunt trauma and injury from the tethered soft tissue. According to a cadaveric study by Bennett and Sisto [19], the safe interval is between the lateral collateral ligament and the anterior part of biceps femoris for this portal. From our experience, creating the posterolateral portal with the point that posterior cortex of fibula intersected with the joint line of knee is also an easy and safe way.

In conclusion, the patients presenting posterolateral pain, fabella syndrome cannot be ignored due to its relative higher presence in Asian population. In the same time, it also requires thorough physical examination, radiography, sonography, even MRI to rule other possible pathologies. The surgical excision can be considered if the non-operative treatment was not able to relief the symptoms. In our experience, the all-arthroscopic fabellectomy offers a smaller wound size, less post-operative pain, fewer days of hospitalization and quicker time to rehabilitation for the patients with chronic posterolateral knee pain caused by fabella syndrome.

## Data Availability

The datasets used and/or analysed during the current study are available from the corresponding author on reasonable request.

## Abbreviations

MRI: Magnetic Resonance Imaging
TKA: Total knee arthroplasty
rESWT: Radial extracorporeal shock wave therapy
WOMAC: Western Ontario and McMaster Universities Osteoarthritis Index

## References

[1] Driessen A, Balke M, Offerhaus C (2014). The fabella syndrome - a rare cause of posterolateral knee pain: a review of the literature and two case reports. BMC Musculoskelet Disord.

[2] Berthaume MA, Di Federico E, Bull AMJ (2019). Fabella prevalence rate increases over 150 years, and rates of other sesamoid bones remain constant: a systematic review. J Anat.

[3] Berthaume MA, Bull AMJ (2020). Human biological variation in sesamoid bone prevalence: the curious case of the fabella. J Anat.

[4] Dekker TJ, Crawford MD, DePhillipo NN (2020). Clinical presentation and outcomes associated with Fabellectomy in the setting of Fabella syndrome. Orthop J Sports Med.

[5] Hou W, Xu L, Wang J (2019). Fabellar prevalence, degeneration and association with knee osteoarthritis in the Chinese population. Sci Rep.

[6] Legendre PFJ, Godin C. Chondromalacia of the fabella: a case report. Can J Surg. 1986;29(2):102–3. 3955458

[7] English SPD. Posterior knee pain. Curr Rev Musculoskelet Med. 2010;3(1–4):3–10. 10.1007/s12178-010-9057-4 PMC2941578 21063493

[8] Provencher MT, Sanchez G, Ferrari MB (2017). Arthroscopy-assisted Fabella excision: surgical technique. Arthrosc Tech.

[9] Dannawi Z, Khanduja V, Vemulapalli KK, Zammit J, El-Zebdeh M (2007). Arthroscopic excision of the Fabella –a report of two cases. J Knee Surg.

[10] Kawashima T, Takeishi H, Yoshitomi S, Ito M, Sasaki H (2008). Anatomical study of the fabella, fabellar complex and its clinical implications. Surg Radiol Anat: SRA.

[11] Chew CP, Lee KH, Koh JSB, Howe TS (2014). Incidence and radiological characteristics of fabellae in an Asian population. Singap Med J.

[12] Egerci O, Kose O, Turan A, Kilicaslan O, Sekerci R, Keles-Celik N (2017). Prevalence and distribution of fabella; a radiographic study in Turkish subjects. Folia Morphol (Warsz).

[13] Pop TS, Pop AM, Olah P, Trambitas C (2018). Prevalence of the fabella and its association with pain in the posterolateral corner of the knee: a cross-sectional study in a Romanian population. Medicine (Baltimore).

[14] Tabira Y, Saga T, Takahashi N, Watanabe K, Nakamura M, Yamaki K-I (2013). Influence of a fabella in the gastrocnemius muscle on the common fibular nerve in Japanese subjects. Clin Anat.

[15] Kimura T, Tanikawa H, Hasegawa T (2019). Late onset of the Fabella syndrome after Total knee Arthroplasty. Case Rep Orthop.

[16] Larson JE, Becker DA (1993). Fabellar impingement in total knee arthroplasty: a case report. J Arthroplast.

[17] Zipple J, Hammer R, Loubert P (2003). Treatment of Fabella syndrome with manual therapy: a case report. J Orthopaed Sports Phys Ther.

[18] Seol P-H, Ha KW, Kim YH, Kwak H-J, Park S-W, Ryu B-J (2016). Effect of radial extracorporeal shock wave therapy in patients with Fabella syndrome. Ann Rehabil Med.

[19] Bennett WF, Sisto D (1995). Arthroscopic lateral portals revisited. A cadaveric study of the safe zones. Am J Orthop (Belle Mead NJ).

